# Design of commercially comparable nanotherapeutic agent against human disease-causing parasite, *Leishmania*

**DOI:** 10.1038/s41598-018-27170-1

**Published:** 2018-06-11

**Authors:** Anupriya Baranwal, Adarsh Kumar Chiranjivi, Ashutosh Kumar, Vikash Kumar Dubey, Pranjal Chandra

**Affiliations:** Department of Biosciences and Bioengineering, Indian Institute of Technology Guwahati, Guwahati, 781039 Assam India

## Abstract

Nanotherapeutic agents (NTA) play a crucial role in clinical medicine, if their unique properties are well understood and well exploited. In this direction, we report synthesis and characterization of highly potent phytofabricated silver nanoparticles (AgNPs) using *Sechium edule*, which served the purpose of both reducing and capping agent. The designed AgNPs were characterized using UV-Vis spectroscopy, XRD, FTIR, HR-TEM, and TGA techniques. The formation of AgNPs was also confirmed using electrochemistry, which to the best of our knowledge has never been reported before for biosynthesized nanoparticles. The antileishmanial potential of AgNPs was examined on the clinical isolates of *Leishmania donovani* promastigote cells in an *in vitro* experimental setting. A dose dependent killing activity of the AgNP was observed with an IC_50_ value of 51.88 ± 3.51 µg/ml. These results were also compared using commercially available drug, miltefosine. Furthermore, the clinical applicability of AgNP, as antileishmanial agent was proven by testing them against normal mammalian monocyte cell line (U937). The results were statistically analyzed and no significant toxicity of AgNPs on the normal mammalian cells was observed.

## Introduction

Leishmaniasis, an emerging disease caused by parasitic protozoan of genus *Leishmania* is amongst one of the most neglected diseases across the globe. So far, 350 million people in 88 tropical and subtropical countries are living under threat of this disease^1,2^. Clinically leishmaniasis is characterized in three different forms *viz*. cutaneous, muco-cutaneous, and visceral form depending on protozoan spp. responsible for its cause^3^. Among all the different forms of this disease, cutaneous is more prevalent; however, effects of visceral form are more life threatening^4,5^. Albeit certain medications are available to control the disease but their high cost, parenteral drug administration (except miltefosine), narrow spectrum effect, and associated severe side effects limit their usage^6^. For instance, pentavalent antimonial, once considered a paradigm of standard drug has become incapable of mitigating the disease due to evolution of resistance in parasitic strains^7^. Miltefosine, another gold standard drug, is incapable of treating visceral leishmaniasis in patients suffering from HIV^8^. Emergence of resistance towards available antileishmanial drugs has brought in the imperative need for the development of new therapeutics that should not only be cost effective, broad spectrum but also safe and within the reach of underprivileged people who are more vulnerable to leishmaniasis.

Nanobiotechnology as a budding field has witnessed outstanding progress in generating nanomaterials and incorporating them for widespread biotechnological applications^9–11^. In recent years, nanomedicine, an anticipated ambitious field of nanobiotechnology, has defined the application of nanomaterials in theranostic domain^12^. Various well established conventional methods are known for synthesizing nanoparticles, however, their usage for biomedical applications is limited due to cellular and environmental toxicity^10,13^. In order to overcome these limitations, biosynthesis has played an indispensable role by offering advantage of being simplistic, non-toxic, benign, and commercially viable green approach to obtain nanoparticles^14^. Owing to exceptional optical and biocompatible properties, AgNPs have found their applications in widespread domains, such as bio-imaging, gene and drug delivery, sensing, and antimicrobial medications to ward off pathogenic microbes^15^. In few reports, AgNPs have been used as a carrier molecule for drugs against some spp. of *Leishmania*^6,8,16^. These reports are interesting; however, the strategy followed requires additional drug conjugation and characterization step which limits its rigorous application in treating leishmaniasis. Therefore, synthesizing biocompatible AgNPs which show comparable antileishmanial activity would be interesting to attempt. It is also, possible to anticipate that these NTAs would be much cheaper compared to the commercially available drugs. Amongst numerous biosynthetic approaches, plant extract mediated synthesis is most preferred because they are readily available; possess vast range of phytochemicals, and allow safe handling. However, there are limited reports on AgNP being phytofabricated and used as NTA for curing leishmaniasis. Development of a standard drug is usually very exhaustive procedure, as it involves multiple steps and takes several years. Moreover, some of these drugs also exert cytotoxicity towards non-target cells and have narrow spectrum effects on parasite^8^. Therefore, phytofabrication could be an approach which may mitigate aforementioned issues and offer a straight forward method to produce highly potent NTA. Inspired by this, we tried to synthesize AgNPs in a simple experimental setting using *S*. *edule* fruit extract which contains several potential reducing and capping agents with an objective to find better NTA against leishmaniasis.

**Figure 1:**
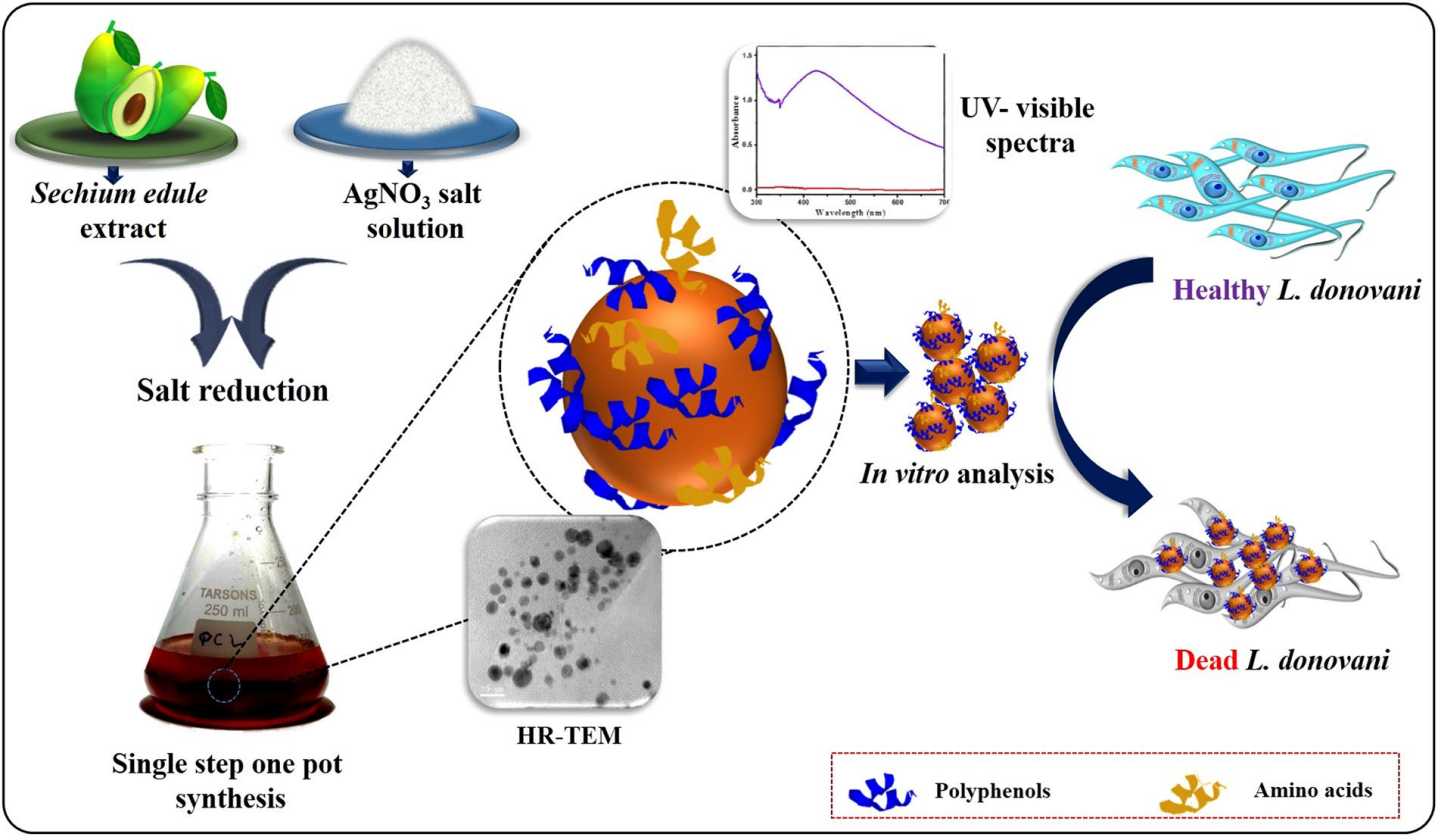
Pictorial representation of single step NTA synthesis and its antileishmanial activity against *L*. *donovani*.

Herein, we propose a systematic procedure to enable facile and economic preparation of AgNPs by using aqueous extract of *S*. *edule* fruit. These AgNPs were then characterized using various techniques such as; UV-Visible spectroscopy, XRD analysis, FTIR spectroscopy, HRTEM imaging, electrochemistry, and TGA. Further, these AgNPs were studied for their antileishmanial potential using clinical isolates of *L*. *donovani*. Schematic representation of NTA preparation and its antileishmanial activity against *L*. *donovani* is shown in Figure 1. The efficacy of the AgNPs was also compared using commercially available drug, miltefosine and their clinical potential were examined by testing them against normal mammalian monocyte cell line (U937). To the best of our knowledge, this is the first report, where *S*. *edule* extract mediated AgNPs were used as a NTA against *L*. *donovani* and the designed nanoparticles were electrochemically characterized which has not been reported so far for any phytofabricated nanoparticles.

## Results and Discussion

### UV-Visible spectroscopy of silver nanoparticles

**Figure 2:**
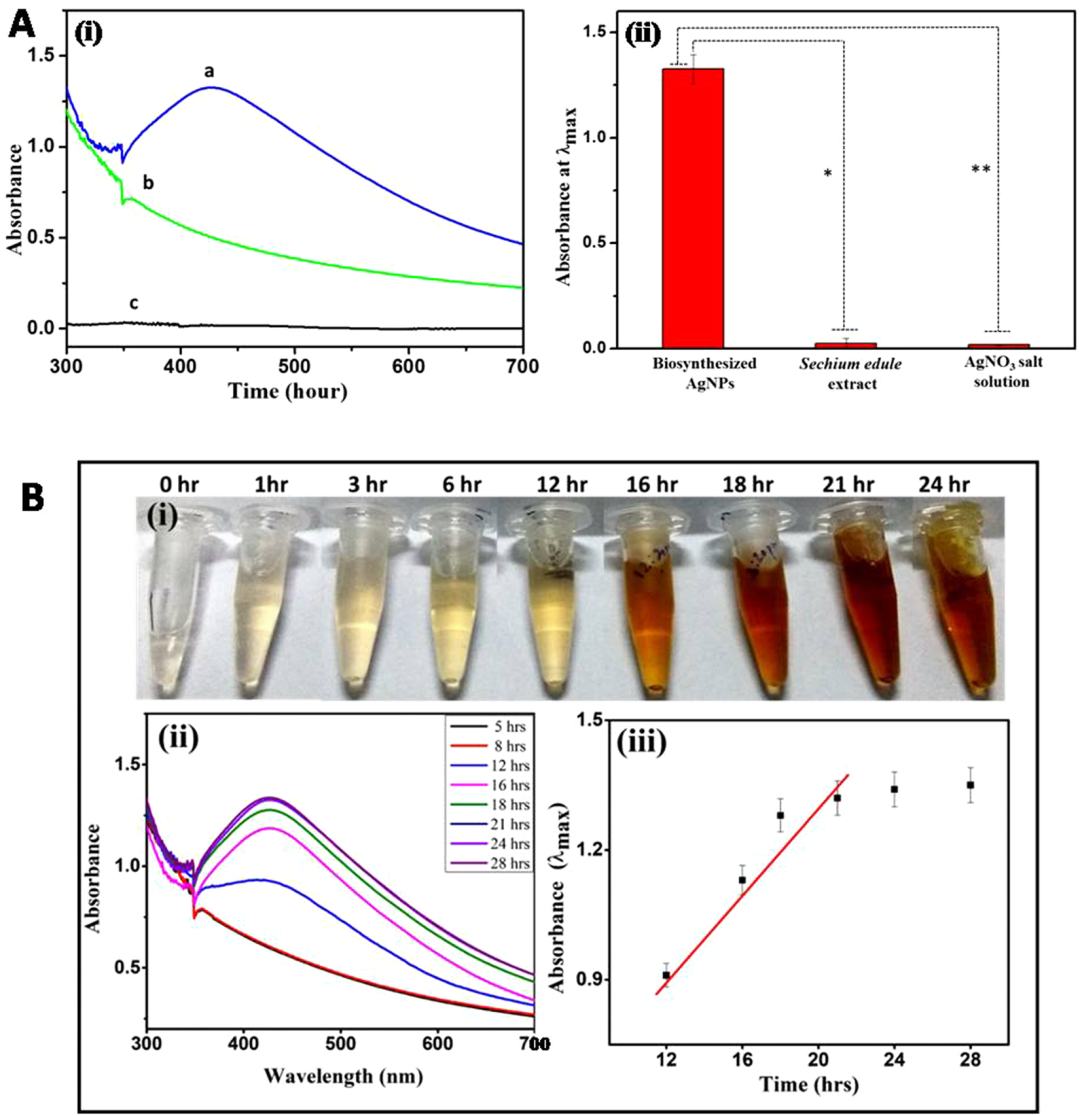
**(A)** (i) UV-Vis absorbance spectra for (a) AgNP biosynthesis, (b) *S*. *edule* extract without AgNO_3_ solution, and (c) AgNO_3_ solution without *S*. *edule* extract, (ii) Histogram corresponding to data shown in (i), the p-values for * and ** were found to be < 0.001, (*n* = *3)*. **(B)** (i) Change in reaction mixture color from transparent to pale yellow to yellowish brown with the progression of incubation time between 0 to 28 hr. (ii) UV-Vis spectra of reaction mixture containing *S*. *edule* extract and aqueous AgNO_3_ as a function of reaction incubation time, and (iii) Linear regression curve analysis for time dependent AgNPs formation.

First indication of the AgNP formation was obtained using UV-Vis spectroscopy in the reaction mixture containing AgNO_3_ and *S*. *edule* extract in equal ratio. Figure 2A(i(a)) shows a representative surface plasmon resonance (SPR) band at 435 nm which indicates preliminary formation of AgNPs. In order to confirm this result, we performed a series of control experiments. In the first experiment, absorbance for *S*. *edule* extract without AgNO_3_ was recorded, where no SPR band at 435 nm was observed as shown in Figure 2A(i(b)). In the second control experiment, AgNO_3_ without addition of *S*. *edule* extract was tested under similar experimental conditions and interestingly again no absorbance was evident at 435 nm (Figure 2A(i(c))). The results obtained based on these control experiments clearly show that the formation of AgNPs in the test sample is only mediated through the *S*. *edule* extract. No signal at 435 nm for AgNO_3_ solution and *S*. *edule* extract proves results to be accurate. We also calculated p-values for * and ** shown in Figure 2A(ii) and the values were found to be <0.001 confirming the statistical significance of data. We further studied the time dependent AgNP formation, where the concentration and volume of AgNO_3_ solution, volume of *S*. *edule* extract, and reaction conditions were kept constant. The change in reaction mixture color from transparent (0 hr) to pale yellow (between 1–5 hr) to yellow (after 12 hr) and finally to yellowish brown was observed after completion of the reaction (Figure 2B(i)). This change in reaction mixture color correspond to variation in the SPR of AgNPs.

The kinetics of AgNP formation was analyzed using UV-Vis spectroscopy and the obtained spectra is shown in Figure 2B(ii). It was observed that with the progression of reaction, a band centered at 435 nm became evident (after 12 hr) which corresponds to the characteristic longitudinal SPR band of AgNPs. This band showed monotonic increase in its absorbance with respect to time without any shift in the wavelength peak. In addition to this band, another band corresponding to transverse SPR of AgNPs was observed at 368 nm, that later disappeared (after 5 hr). These bands are discrete and exhibit a difference of 67 nm in their λ_max_ values that indicates aggregation of initially formed AgNPs in order to give final stable nanoparticles^17^. Reduction of Ag^+^ ions to Ag° in the presence of *S*. *edule* extract seemed fairly rapid as 90% of the reaction was complete within 18 hr, however, after 21 hr of reaction no reduction was evident, probably due to exhaustion of Ag^+^ ions or the phytochemicals which assisted in reduction of Ag^+^ ions in reaction mixture (Figure 2B(ii)). Figure 2B(iii) shows the linear regression plot for time dependent formation of AgNPs between 12–28 hr, where the absorbance increases with the increase in reaction time. The linear regression equation for AgNP formation is expressed as, Abs [λ_max_] = 0.525 (±0.016) + 0.014 (±0.001) [time] with the correlation coefficient of 0.972 which delineates the significance of Figure 2B(ii), justifying that with increase in reaction time, there will be increase in absorbance at λ_max_^18^, however, this relation is only valid till 21 hr because after that no change was observed in absorbance at λ_max_ values. The slope of the calibration plot was found to be 0.014 with RMSD <7%, indicating highly reproducible synthetic procedure of AgNP synthesis.

### XRD analysis

**Figure 3:**
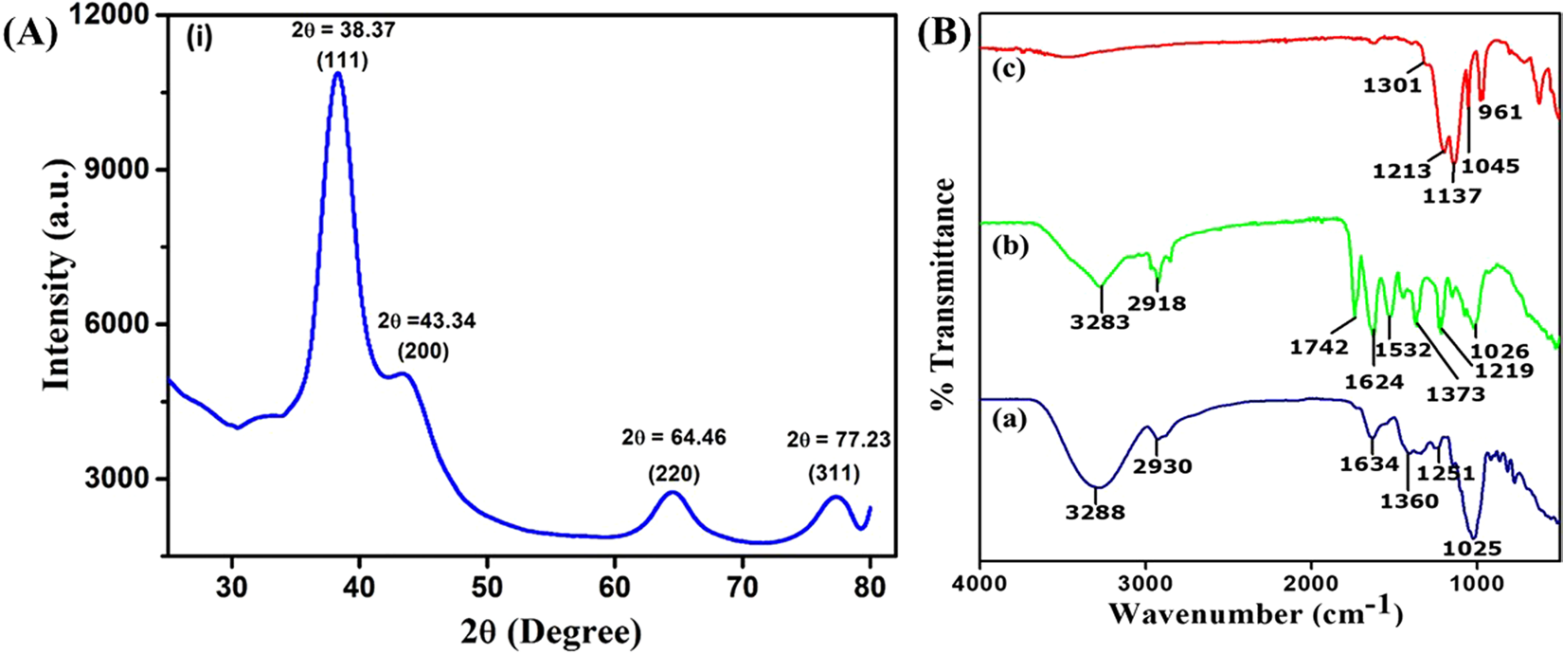
**(A)** X-ray diffractogram of biosynthesized AgNPs. **(B)** The FTIR analysis of (a) *S*. *edule* fruit extract, (b) AgNPs obtained by adding equal volumes of AgNO_3_ salt solution and *S*. *edule* fruit extract, and (c) chemically synthesized AgNPs.

The XRD analysis of AgNPs was carried out in between a range of 2θ values from 30° to 80°. It generated a diffraction pattern as shown in Figure 3A, where four major peaks were observed at 38.37°, 43.34°, 64.46°, and 77.23°. These peaks were observed due to reflection of X-rays from different crystal planes (111), (200), (220), and (311) in a respective order and the obtained spectra indicates the face centered cubic (fcc) structure of AgNPs (JCPDS reference file no. 65–2871). As it is evident from the diffractogram, the most significant peak was observed at 2θ = 38.37° which clearly indicates that the topmost crystal plane i.e., basal plane for AgNPs must be the (111) crystal plane. The broad peaks observed in diffraction pattern signify presence of smaller size particles and effect of experimental conditions on the nucleation and crystal nucleus^19^. The crystallite size (D) of AgNPs was calculated by using Debye – Scherrer equation (1)

1 $$D=\frac{k\lambda }{\beta \,\cos \,\theta }$$

where, D is crystallite size in nm, K is Scherrer’s constant with its value ranging from 0.9 to 1, λ is X- ray source wavelength, β is diffraction line broadening measured in radians at half of the maximum intensity i.e., full width half maxima (FWHM), and θ is Bragg’s angle of diffraction in degrees. The crystallite size calculated for AgNPs was found to be ca. 3.4 nm. This result was found to be in agreement with the HR-TEM imaging analysis for average nanoparticle size discussed in the upcoming section.

### FTIR analysis

Further, we characterized the AgNPs using FTIR spectroscopy in order to understand the involvement of phytochemicals including macromolecules like proteins, carbohydrates, etc. in their formation. Figure 3B shows the representative FTIR spectra of *S*. *edule* extract (a) and biosynthesized AgNPs (b). For sample (a) the broad peak at 3288 cm^−1^ can be assigned to -OH stretch of alcohols and phenolic compounds, 2930 cm^−1^ to C-H stretch of alkanes, similarly 1634 cm^−1^, 1360 cm^−1^; 1251 cm^−1^ and 1025 cm^−1^ can be assigned to C = O stretch of amides, CH_3_C-H bend of alkanes, and = C-O-C symmetric stretch of aromatic amides, -CH_3_C-H bend of alkanes, and = C-O-C symmetric stretch of aromatic ethers, C-O stretch of aromatic alcohol, respectively^20^. Likewise, for sample (b) the 3283 cm^− 1^ band can be attributed to -OH stretch of alcohols and phenolic compounds, 2918 cm^−1^ to C-H stretch of alkanes, 1742 cm^− 1^ to -C = O stretch of esters, 1624 cm^−1^ to N-H bend of amines, 1532 cm^−1^ and 1373 cm^−1^ to N-O symmetric and asymmetric stretch of nitro compounds; and 1219 cm^−1^ and 1026 cm^−1^ to =C-O-C symmetric and asymmetric stretch of aromatic ethers. A few variations in the form of peak shift, peak disappearance, and appearance of new peaks were observed in FTIR spectra for the phytofabricated AgNPs. The shift in bands was observed from 3288 to 3283 cm^−1^, 2930 to 2918 cm^−1^, 1634 to 1624 cm^−1^, 1360 to 1373 cm^−1^, 1251 to 1219 cm^−1^, and 1025 to 1026 cm^−1^ which is most likely due to the involvement of phytochemicals in reduction of silver ions and stabilization of AgNPs due to their capping. It is interesting to note the that two new bands 1742 cm^−1^ and 1532 cm^−1^ which correspond to C=O stretch and N-O symmetric stretch, respectively appeared for sample (b). We anticipate that the appearance of 1742 cm^−1^ band could be due to oxidation of aromatic alcohol (e.g. polyphenols) into C=O containing aromatic compound (e.g. Quinones). It has been reported that *S*. *edule* fruits are rich in flavonoids (polyphenols)^20,21^, which most likely assisted the reduction of Ag^+^ ions into Ag° in this case, eventually forming AgNPs. We also anticipate that the reduction of amino acids (aspartic acid, glutamic acid, tyrosine, etc.) present in *S*. *edule* fruit extract^20^ resulted in the formation of nitro amine compound i.e., band at 1532 cm^−1^ which eventually assisted in AgNP stabilization. The involvement of the compounds present in fruit extract in the formation of AgNPs was also confirmed by an additional control experiment, where AgNPs were synthesized by using an earlier reported chemical reduction method^22,23^. The FTIR analysis spectrum obtained for chemically synthesized AgNPs, sample (c) is shown in Figure 3B. Interestingly, the peaks observed in the case of sample (c) (red curve) lacks those representative peaks (*n* = *5*) which appeared in case of biosynthesized AgNPs, sample (b) (green curve). The peaks observed at 1301 cm^−1^ and 1137 cm^−1^ can be assigned to S=O stretch, peak at 961 cm^−1^ to S-O stretch, and 1045 cm^−1^ to C-F stretch. These characteristic peaks correspond to the functional groups present in nafion^24^ which in this case assisted in the stabilization of chemically synthesized AgNPs. This clearly suggests that the peaks observed in case of sample (b) are merely due to compounds present in the *S*. *edule* fruit extract.

### HR-TEM imaging analysis

**Figure 4:**
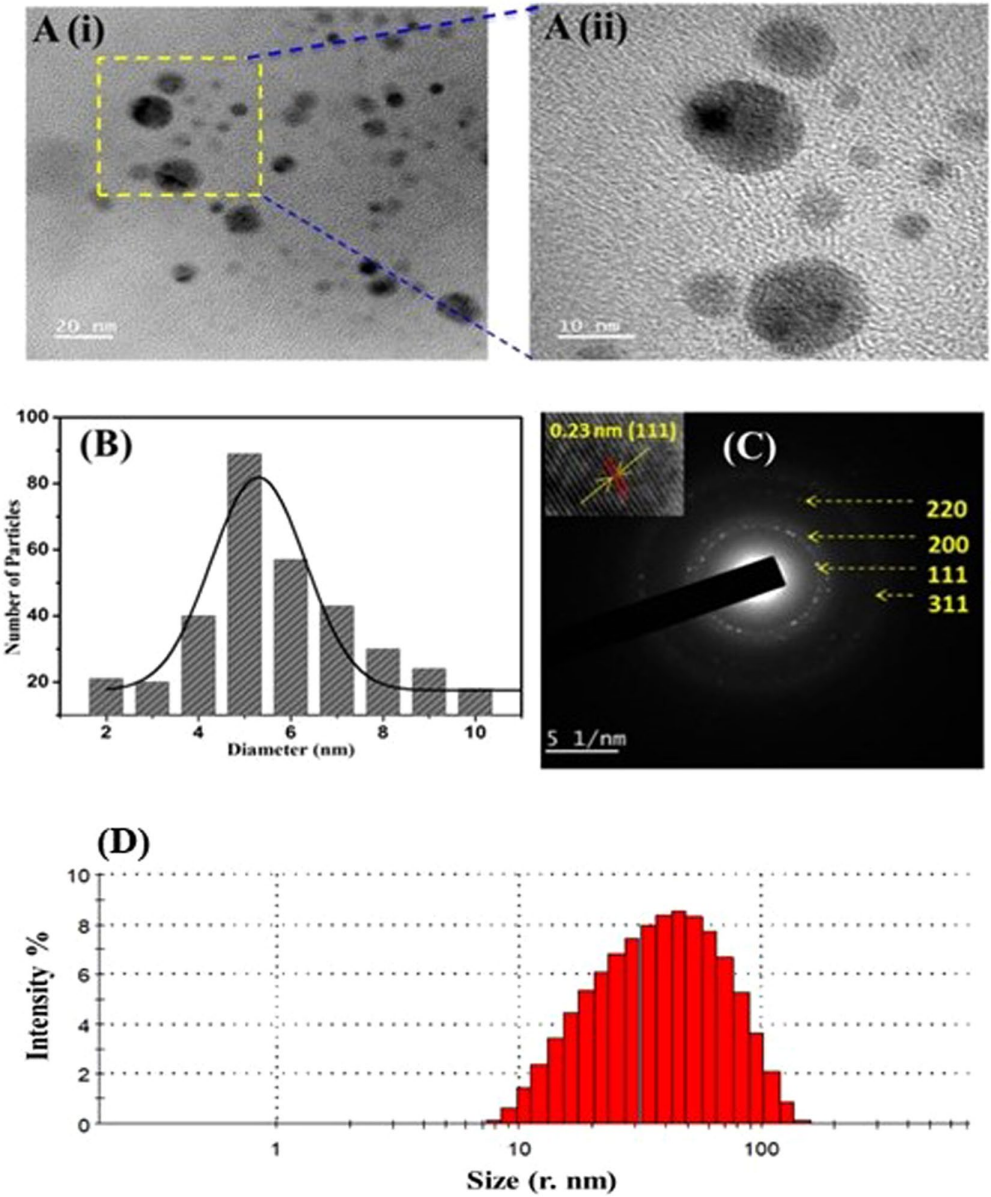
**(A)** The representative HR-TEM images of AgNPs after completion of reaction at different magnification scales (i) 20 nm and (ii) 10 nm, **(B)** average particle size distribution of prepared AgNPs, **(C)** SAED pattern showing crystalline planes for fcc structure of AgNPs, inset shows fringe distance, and **(D)** Size distribution analysis of AgNPs observed via. dynamic light scattering.

The AgNP size, its morphology, and dispersity were analyzed via. HR-TEM imaging which is shown in Figure 4. Determination of these parameters is highly recommended as they regulate the biological properties to be associated with AgNPs. It is evident from Figure 4(A(i)), that at low magnification (scale 20 nm) AgNPs appeared in dense form, revealing synthesis of polydisperse AgNPs of size ranging from 2 to 10 nm. However, at high magnification (scale 10 nm) Figure 4(A(ii)), their morphology became more conspicuous. Most particles exhibited exclusive spherical shape with a few having nearly spherical structure. Figure 4(A) also shows that the synthesized AgNPs are in well dispersed state, thus signifying negligible nanoparticle aggregation after completion of reaction which could be due to the capping molecules present in *S*. *edule* extract. Further, the average particle size distribution (Figure 4(B)) was calculated using ImageJ software which confirms the average AgNP size to be 5 ± 2 nm. It is interesting to note that the size obtained in our case is much lower compared to the previous reports published on phytofabricated AgNPs^25,26^, and possess comparable size when prepared using chemical methods^23,27^. The minor size difference ca. 1 nm observed in XRD and HR-TEM is possibly due to the difference in sample preparation methodologies and the analysis principles^19^. Figure 4(C) depicts the selected area electron diffraction (SAED) pattern, in which crystallite nature of AgNP is evident. On the basis of silver fcc structure, indexing of diffraction points in the SAED pattern can be attributed to (111), (200), (220), and (311) planes, respectively and this finding is in agreement with the XRD data.

### DLS analysis

The hydrodynamic radius and the dispersity of AgNPs were analyzed using DLS which is shown in Figure 4(D). It is clearly evident from this figure that the size distribution of AgNPs ranges between 10 to 100 nm which signifies that the synthesized nanoparticles are polydispersed in nature. The calculated average particle size distribution of AgNPs was found to be 50.62 nm. The difference between AgNPs sizes calculated from HRTEM and DLS is due to the fact that DLS gives hydrodynamic radius which is the size of a hypothetical hard sphere that diffuses in the same fashion as that of the particle under study^28^. However, HRTEM gives information based on the electron rich part which is actually the inner core of the particle and hence, the smaller size is obtained.

### EC analysis

**Figure 5:**
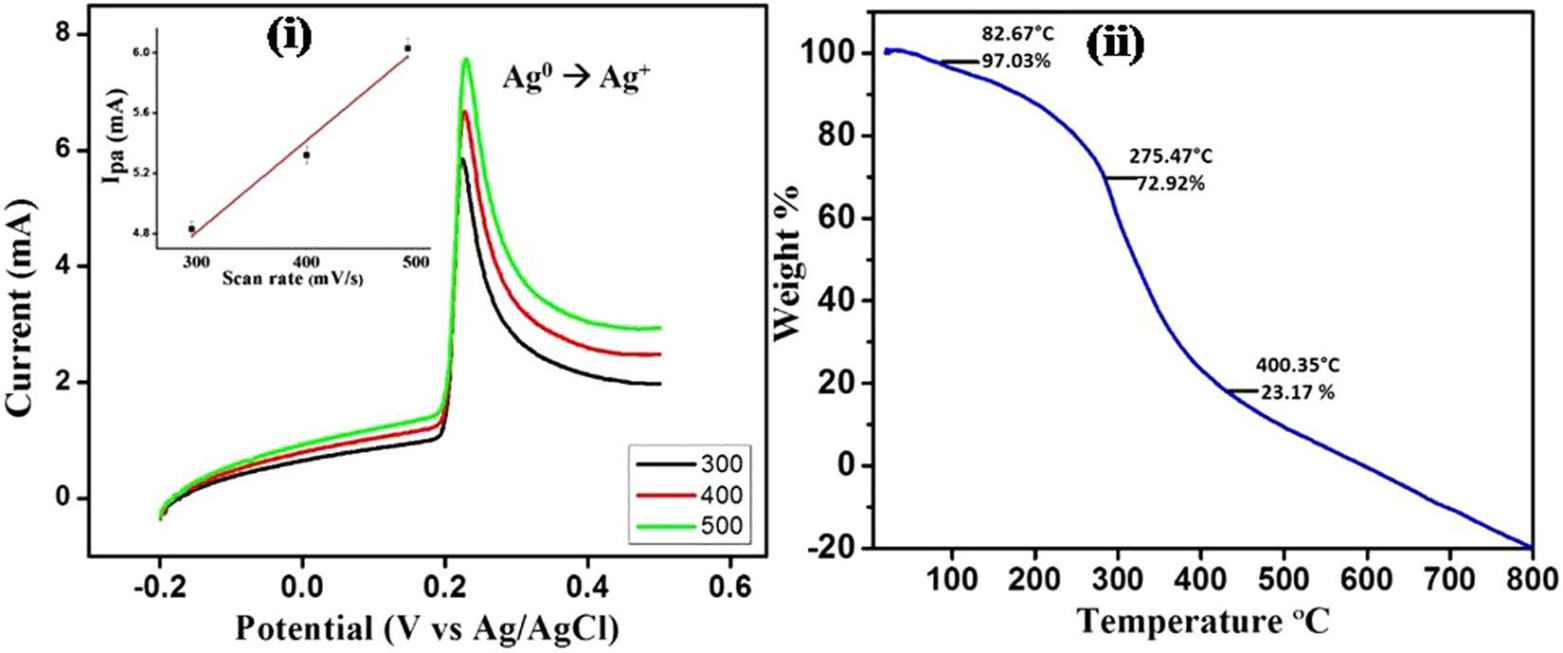
(i) LSV responses on different scan rates of 300, 400, and 500 (mV/s) at modified GC electrode (GCE/AgNPs/Naf) in 0.1 M PBS; Inset figure shows the linear relation between anodic peak current and different scan rates, (ii) Thermal gravimetric analysis curve of AgNPs.

We also confirmed the synthesis of AgNPs using linear sweep voltammetry (LSV). For this purpose, the nafion modified glassy carbon electrode (GCE/AgNPs/Naf) surface was used as working electrode. Figure 5(i) shows the representative LSV, where a sharp oxidation peak at +0.21 V *vs*. Ag/AgCl was observed due to the oxidation of AgNPs to Ag^+^ (Ag^0^→Ag^+^) (black curve). In order to confirm that the peak at +0.21 V arose merely due to Ag^0^→Ag^+^, we recorded LSVs with increase in the scan rates between 300 and 500 mV/s Interestingly, the peak currents were directly proportional to scan rates, indicating the surface confined electrochemical process due to AgNPs attached onto the GCE surface. A linear plot for AgNPs oxidation with scan rates was obtained having the regression equation as follows: Ipa = 2.996 (±0.253) + 0.005 (±0.006) [scan rate], with a correlation coefficient of 0.978 *(n* = *5)*, indicating high stability of the synthesized nanoparticles. This was also confirmed by performing a negative control experiment, where bare GCE was scanned under the similar potential window under similar experimental conditions. In this case, no peaks were obtained even at higher scan rates, indicating peaks in the earlier case are merely due to the oxidation of AgNPs (Ag^0^→Ag^+^) attached onto the electrode surface. It is worthy to mention here that, this is the first report where an electrochemical method has been applied for precise and ultrafast (<30 s) characterization of the AgNPs which has not been reported so far. These results may pave way towards application of this method for quick characterization of other metallic nanoparticles prepared using biological processes.

### TGA analysis

After characterizing the AgNPs by various aforementioned techniques, we further studied their thermal stability using TGA (Figure 5(ii)). A ceramic alumina crucible was used for raising the temperature of powdered AgNPs and TGA data was recorded between a temperature range from room temperature to 800 °C. It is evident from the TGA curve that 97.03% weight of AgNPs was retained until 82.6 °C. The slight weight loss observed in here was presumably due to loss of moisture present in the nanoparticles^29^. The predominant loss in AgNPs’ weight started from 200 °C and continued till 450 °C, however, complete weight loss was observed at 500 °C. The overall TGA analysis clearly shows that these AgNPs are reasonably stable up to 80 °C, suggesting their potential application in photo-thermal therapy as NTA^30^. However, in order to develop a successful NTA other properties and factors should also be considered.

### Evaluation of antileishmanial activity and cytotoxicity of silver nanoparticles

In order to study the biomedical implication of designed AgNPs, its effect on *L*. *donovani* strain isolated from clinical samples was studied using MTT assay. The result of the MTT assay is presented in the form of percentage cell viability which was calculated by using equation (2):

2 $$ \% Cell\,viability=\frac{Mean\,O{D}_{570}of\,test\,samples}{Mean\,O{D}_{570}of\,control}\times 100$$

**Figure 6:**
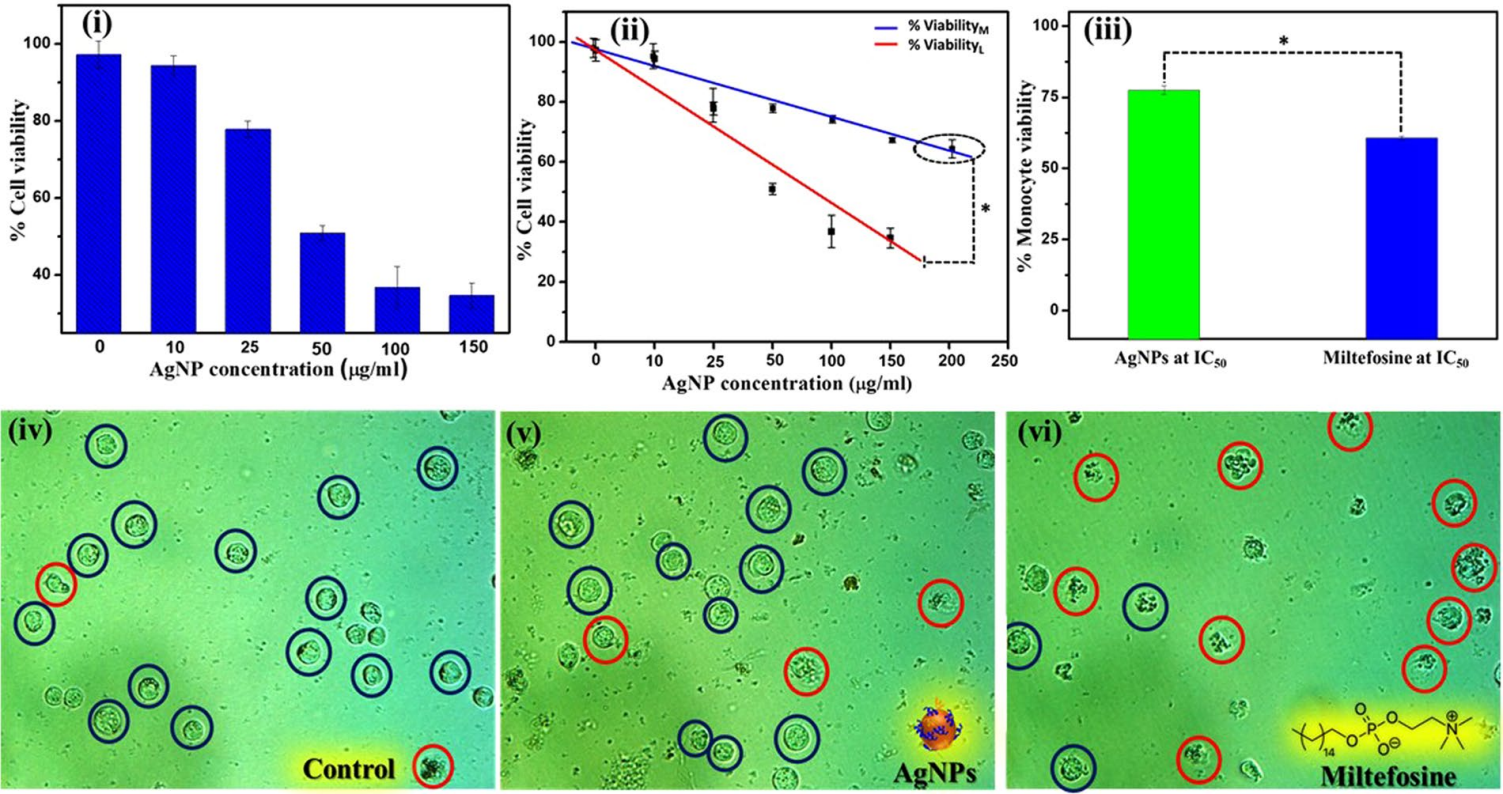
(i) Impact of varying concentrations of phytofabricated AgNPs on *L*. *donovani* viability. (ii) Cytotoxicity assessment of AgNPs shows the relation between parasite (red curve) and monocyte (blue curve) viability as a function of AgNP concentration (p- value for * was < 0.0001, *n* = *3*). (iii) Comparative analysis of the % viability of monocyte at IC_50_ values of AgNP and Miltefosine (p- value for * was < 0.0001, *n* = *4*). Microscopic images of monocyte cell line observed at 40X magnification (iv) negative control (untreated cells), (v) test sample (cells treated with AgNPs at IC_50_ concentration), and (vi) positive control (cells treated with miltefosine at IC_50_ concentration). The blue circle shows healthy monocytes and red circle shows deformed, ruptured, and dead cells.

For this purpose, firstly the cytotoxic effect of 100 µg/ml AgNP was tested against the parasite promastigotes. The percentage cell viability observed in this case was 35.21 ± 4.03% *(n* = *3)*, indicating significant cytotoxic effect of AgNPs. In order to confirm this phenomenon, we evaluated AgNP’s cytotoxicity over a range of concentrations, from 10 to 150 µg/ml. It was interesting to note, that with increase in AgNPs concentration there was a decrease in percentage viability of *L*. *donovani*, suggesting a dose dependent killing action of AgNPs (Figure 6(i)). A caliberartion plot delineating dose dependent cytotoxic effect of AgNPs has been shown in Figure 6(ii). The linear regression equation for this plot is expressed as follows: % Viability_L_ = 101.098 (±2.778) −0.992 (±0.074) [AgNP] with the correlation coefficient of 0.983 and RMSD <5.41%, (*n* = *3)* indicating a highly reproducible data in terms of the cytotoxic effect of AgNPs. To confirm the detreimental effect of AgNP, a negative control experiment was performed where cells were not treated with AgNPs. In this case the percentage cell viability was 98.10 ± 2.2% (*n* = *3*), indicating that the cell death in the earlier case was merely due to the killing effect of AgNPs. Further, we evaluated the IC_50_ value of AgNPs which was found to be 51.88 ± 3.51 µg/ml (*n* = *3*). It is worthwhile to discuss that the IC_50_ value obtained in our case is comparable with the IC_50_ value of chemically synthesized AgNPs which has been assayed against *L*. *tropica*^31^. This indicates that the phytofabricated AgNPs synthesized in our case have similar potency to that of chemically synthesized nanoparticles and thus, they can be used as a powerful therapeutic agent.

For most of the NTAs, the major challenge is their cytotoxicity against normal mammalian cells, which is not acceptable and eventually those NTAs can not be used for biomedical application in a real sense^32,33^. In the view of this important issue, we further investigated the effect of AgNPs on human monocyte U937 (normal cells) (Figure 6(ii) blue curve). For this purpose, these monocytes were treated with AgNPs at a concentration range similar to that used for *L*. *donovani* promastigotes in order to compare their effect on both the cells statistically. A dose dependent decrease in the percentage viability was observed in this case too, however it was singificantly higher compared to that observed in the case of *L*. *donovani* (Figure 6(ii) red curve). The linear regression equation for percentage viability of monocyte is expressed as follows: % cell viability_M_ = 96.967 (±2.942) −0.608 (±0.066) [AgNP] with the correlation coefficient of 0.942 and RMSD <5.24%, (*n* = *3*). Further, to confirm that AgNPs have no toxic effect on monocytes, we tested their effect even at a higher concentration of 200 µg/ml (encircled point in Figure 6(ii) blue curve). The result obtained was encouraging, as the percentage viability in this case is almost similar to that observed when 150 µg/ml of AgNPs were tested, indicating that even at higher concentration these particles do not show any further decrease in monocytes’ viability.

Furthermore, we also compared the percentage monocyte viability at the IC_50_ values of AgNPs and miltefosine, a widely used antileishmanial drug Figure 6(iii). It is noteworthy to witness that the percentage viability of monocyte against AgNP was 78.12 ± 2.21% (*n* = *4*) whereas, for miltefosine it was merely 59.02 ± 1.12% (*n* = *4*) (p-value < 0.0001). This result was also confirmed by microscopic imaging of the monocytes after AgNPs treatment with suitable positive (miltefosine) and negative controls (untreated). It is evident from the bioimaging results that the number of dead cells (red circle) after AgNPs treatment (Figure 6(v)) at its IC_50_ value are close to the negative control (Figure 6(iv)), however, in case of positive control (Figure 6(vi)) the dead cells are relatively higher in number. This observation is in well agreement with the data shown in Figure 6(iii), indicating the accuracy of our method.

Altogether, these results clearly indicate that the designed AgNPs are safe as well as specific for killing *L*. *donovani* parasite isolated from clinical samples. Thus, this work might claim to provide a method to produce highly effective NTA against *L*. *donovani* with less toxicity against normal cell line, which is one of the most important criteria for commercial viability of any NTA. Further, the biochemical studies are underway to understand the detailed molecular mechanism of the antileishmanial effect of the synthesized AgNPs. In future, the biosynthetic method described herein, can be used in pharmaceutical industries for scaled up synthesis of NTA which can be used as a potent antileishmanial agent.

## Experimental Section

### Materials

Silver nitrate (AgNO_3_) was procured from SRL India and fresh *S*. *edule* fruit was purchased from market complex at Indian Institute of Technology Guwahati, Assam, India. Sodium phosphate monobasic, sodium phosphate dibasic, and potassium chloride for PBS preparation; sodium bicarbonate, 4-(2-hydroxyethyl)-1-piperazineethanesulfonic acid (HEPES), M199 media, RPMI-1640 media, 3-(4,5-dimethyethiazol-2-yl)-2,5-diphenyltetrazolium bromide (MTT) dye, miltefosine, and penstrep antibiotic required for parasite culture were supplied by Sigma-Aldrich, USA. Fetal bovine serum (FBS), antibiotic and antimycotic solution for mammalian U937 cell line, and dimethyl sulphoxide (DMSO) was provided by HiMedia, India. The *L*. *donovani* (BHU 1081) strain was provided by Prof. Shyam Sundar, Banaras Hindu University, India and monocyte (U937) cell line was procured from National Centre for Cell Science, Pune, India. Deionized water from Milipore unit (Elix, USA) at 18.2 MΩ was used to prepare all the reagents and solutions.

## Methods

### Phytofabrication of silver nanoparticles

Fresh *S*. *edule* fruit was rinsed twice with tap water followed by deionized water to remove unwanted dirt and epiphytes. 100 g of fruit was roughly cut into large chunks after removing its peel and blended into fine paste. 120 ml of deionized water was added and the paste was centrifuged at 5000 rpm for 5 minutes. The supernatant was filtered using Whatman filter paper (grade 1) and the clear filtrate was stored at 4 °C for further use. Equal volumes (100 ml) of fruit extract and 1 mM aqueous silver nitrate solution were mixed and allowed to react at various temperatures under constant stirring (Spinot, Tarsons, India) in dark. The optimized temperature was found to be 90 °C (data not shown). Reaction was continued for 28 hours (optimized) and samples were extracted in between the process to monitor the progression of reaction with time by measuring absorbance in UV-Vis spectrophotometer. Once, the nanoparticle synthesis was complete, reaction mixture was centrifuged (Sorvall Legend XTR, Thermo Fisher) at 4500 rpm for 20 minutes and the pellet was washed thrice with de-ionized water and ethanol successively. Post centrifugation pellet was dried at 75 °C (overnight) in a hot air oven (Labline) and stored in air tight vessel.

### Characterization of phytofabricated silver nanoparticles

#### UV- Visible (UV-Vis) spectroscopy analysis

UV-Vis spectrophotometer (Carry 100 Bio) was used to determine the bio-reduction process of silver ions (Ag^+^) present in reaction mixture into AgNPs (Ag^0^) by scanning the aliquots from 300 to 700 nm wavelength range at different time intervals.

#### X-ray diffraction (XRD) analysis

The crystallite nature of the AgNPs was determined by using a powder X-ray diffractometer (MiniFlex 300/600), where, XRD analysis was performed at 2θ Bragg’s angle by maintaining the range between 30° to 80° with a scan rate 3° per minute.

#### Fourier transformation infra-red spectroscopy (FTIR) analysis

The FTIR analysis of *S*. *edule* extract, biosynthesized AgNPs, and chemically synthesized AgNPs was carried out with Perkin Elmer FT-IR spectrometer Spectrum Two and data was recorded between wavenumber ranging from 500–4000 cm^−1^.

#### Dynamic light scattering (DLS)

The DLS analysis of phytofabricated AgNPs was performed by using Beckman Coulter: Delsa Nano C to analyze their hydrodynamic radius and dispersity of the synthesized nanoparticles.

#### High resolution Transmission electron microscopy (HR-TEM) imaging analysis

Shape, size, and dispersity analyses of phytofabricated AgNPs were performed with HR-TEM (JEOL JEM-2100F TEM). Samples were prepared by drop casting the well dispersed AgNPs (purified by centrifugation) onto the surface of copper grid (200 mesh) and allowing it to dry at 37 °C for 2–3 hrs.

#### Electrochemical (EC) analysis

EC analysis of prepared AgNPs was carried out by sweeping the potential in LSV between −0.2 and 0.5 V *vs*. Ag/AgCl using a potentiostat/galvanostat (Kosentech, Model: KST P-2) in 0.1 M PBS. In this case, the AgNPs-Naf modified glassy carbon electrode (GCE) (area 0.07 cm^2^), Ag/AgCl (in saturated KCl), and a platinum (Pt) wire were used as working, reference, and counter electrodes, respectively.

#### Thermal gravimetric analysis (TGA)

Thermal stability and the weight loss analysis of synthesized AgNP was carried out using TGA (*Netzsch*, STA449F3A00) and result was shown in the form of temperature vs. percent weight loss plot.

#### *In vitro* culturing of L. donovani and monocyte

The clinical isolates of *L*. *donovani* (BHU 1081) obtained from a patient suffering from visceral leishmaniasis was cultured using procedure reported in our earlier publications^34,35^. The cells were cultured in M199 medium, supplemented with all essential nutrients, such as sodium bicarbonate salt, penstrep antibiotic and heat inactivated 15% FBS to enable proper growth of promastigotes. T25 (Thermo, India) culture flasks with filter cap were used to allow parasitic growth and these flasks were maintained at 25 °C in a BOD incubator (Scigenics Orbitek). Promastigote cultures were instigated at 2 × 10^6^ cells/ml and allowed to grow for 3 to 4 days after which they were sub-cultured under similar conditions. These cultures were regularly analyzed under inverted microscope (Motic, BA210E Upright) for their health and number and once the media was consumed it was replenished by the fresh media.

Monocyte U937 mammalian cell line was used as a cellular target to investigate the cytotoxicity of AgNPs against normal cells under *in vitro* conditions. These cells were maintained at 37 °C in a 95% humid atmosphere and 5% CO_2_ in a CO_2_ incubator (Nectarnova, EPI65ARS) and cultured in RPMI media supplemented with sodium bicarbonate salt, 15% heat inactivated FBS, antibiotic and antimycotic solution, etc. Similar to parasite cultures, these cells were also checked for their health and confluency under inverted microscope.

#### *In vitro* cytotoxicity analysis of phytofabricated silver nanoparticles

The cytotoxicity analysis was done using MTT assay^36,37^ and for this purpose, *L*. *donovani* was used as model microorganism. Briefly, promastigotes were cultured in T25 tissue culture flasks and maintained at 25 °C in an incubator. These cells were then seeded in distinct wells containing 100 μl of M199 media at a final density of 2 × 10^6^ cells/well in a 96 well microtiter plate. After overnight incubation, the cells were treated with miltefosine (IC50~25 µM), as positive control, untreated cells were used as negative control, and cells treated with NTA i.e., AgNP as test sample in separate experiments. The treated cell samples were incubated overnight at 25 °C and all the experiments were performed in triplicates. Post incubation, cells in microtiter plate were centrifuged (Sigma 3–30 K) at 4500 rpm for 45 minutes and media was discarded to obtain the cells in the form of pellet. After centrifugation, 180 μl of MTT reagent (0.5 mg/ml) was added into each well and the plate was incubated again at 25 °C in dark for 4 hrs. After this, MTT was washed off and the formazan crystals were solubilized in DMSO (100 μl/well). Reduction of MTT was measured by recording the absorbance at 570 nm by using a microtiter plate reader (BioTek Synergy HT).
